# Gait dataset of 14 Syrian above-knee amputees and 20 healthy subjects

**DOI:** 10.1016/j.dib.2021.107365

**Published:** 2021-09-13

**Authors:** Rufaida Hussain, Zouheir Marmar

**Affiliations:** Damascus University-Biomedical Engineering Department, Damascus University, Syria

**Keywords:** Clinical gait analysis, Above knee amputee, Gait kinematics, Gait kinetics, Spatio-temporal parameters

## Abstract

This Gait dataset is essential for studying motion, evaluating prosthesis effects, and improving the design of prosthetic components to meet the needs for daily life in low-income countries where advanced prosthesis are limited. This dataset contains the gait parameters of 14 above-knee prosthesis users (5 female, 9 male) with different passive prosthetic components and 20 healthy participants (10 male, 10 female). The data are spatio-temporal parameters, lower limb joints angles and moments in the sagittal plane, and ground reaction force components. These data were acquired using 6 video cameras (BTS SMART-D) and 2 Kistler force plates in the biomechanics lab at Damascus University. The acquisition process of data follows Davis et al. protocol for clinical gait analysis. It was divided into three main steps: subject preparation, raw data acquisition, and gait parameters calculation. The online repository containing the files is Mendeley Data: [http://dx.doi.org/10.17632/k5y9jkx87y.1]

## Specifications Table

**Table:** 

Subject	Biomedical Engineering
Specific subject area	Clinical gait analysis of individuals using passive above knee prostheses beside a control group for comparing
Type of data	Tables and figures
How data were acquired	Clinical gait analysis was performed using the optoelectronic system with passive markers (BTS SMART_D, BTS, Milan, Italy) that consists of six cameras and two force platforms (9281EA kistler, Switzerland). All devices acquired data at a sampling rate 200 Hz. The 22 markers were placed according to Davis Heel protocol.
Data format	Analyzed
Parameters for data collection	Gait spatio-temporal parameters, lower limb joints angles and moments in the sagittal plane, and ground reaction force components. They were calculated based on data reduction described by [1]
Description of data collection	Three main steps: 1. subject preparation: subject information and body measuring were taken, then retro-reflective markers were adhesive on her/his body. 2. raw data acquisition: data were acquired at standing (one trial) and walking (3–5 trials) 3. gait parameters calculation: calculated based [1],then the average of three trials of walking were taken as values for gait parameters.
Data source location	Institution: Biomechanics Lab at Biomedical engineering Department- Mechanical and Electrical Engineering Faculty -Damascus University. City: Damascus Country: Syria
Data accessibility	In a public repository Repository name: Mendeley Data Direct URL to data: http://dx.doi.org/10.17632/k5y9jkx87y.1 https://data.mendeley.com/datasets/k5y9jkx87y/1

## Value of the Data

• This Dataset will improve prosthetic and orthotic designs for many reasons. Firstly, it provides researchers and engineers with gait parameters (Spatio-temporal- kinematics and kinetics of lower limb) that need in different design steps. These data were collected in a low-income country (Syria) by an optoelectronic motion capture system that considers as the golden standard method. It will help researchers to figure the functionality of passive prosthetic components and the effect of the most common prosthetic components combinations on the gait of prosthetic users. This data removes some barriers faced by developing prosthetic and orthotic designs. Because gait analysis facilities are expensive, unavailable, inaccessible, and generate an enormous amount of data that needs processing.
• This dataset benefits both clinical and biomedical engineer researchers. They need reference gait data while analyzing the biomechanics of gait deviations, designing lower limb prostheses or orthoses, and evaluating control strategies using simulation. Besides, it is suitable for various biomechanics studies like comparative studies, symmetry indices between the prosthetic and intact side or between them and normal subjects.
• This dataset is learning material for prosthetics and orthotics or biomechanics courses. It utilizes for example as illustration cases, biomechanics problems, human motion simulation inputs, and reference trajectories for controllers.

## 1 Data Description

The data were grouped to five excel files according to gait parameters (four files) besides, participants' characteristics in File 01 Subjects information.xlsx. In gait parameters files, each value is the average of three gait cycles for the considered side (Left or right in healthy subjects which available in spreadsheets that start with N, or Prosthetic and intact in above knee amputee which available in spreadsheets that start with AMP). gait kinematic and kinetic parameters were normalized to gait cycle duration percentage (from 0–100% of gait cycle) in files listed in Table 2. Dataset files are:

1. File 01 Subjects information.xlsx: *N* sheet contains healthy participant's gender, age in years, height in m, and weight in kg, while AMP sheet contains additional information about amputation and prosthesis: years since amputation, amputated side, cause of amputation, K- level(Medicare Functional Classification Levels (MFCL)) [2], and prosthetic component. Fig. 1 shows passive prosthetic components that used. Prosthetic feet are single axis foot (6 participants), Solid Ankle Cushion Heel SACH foot(3 participants), and 1c10 Terion foot otto bock (5 participants). Prosthetic knee joints were 3R20(7 participants), 3R60(4 participants), 3R78(3 participants) . All participants wore quadrilateral socket with suction suspension except one participant who used suction with pin.

**Fig. 1:**
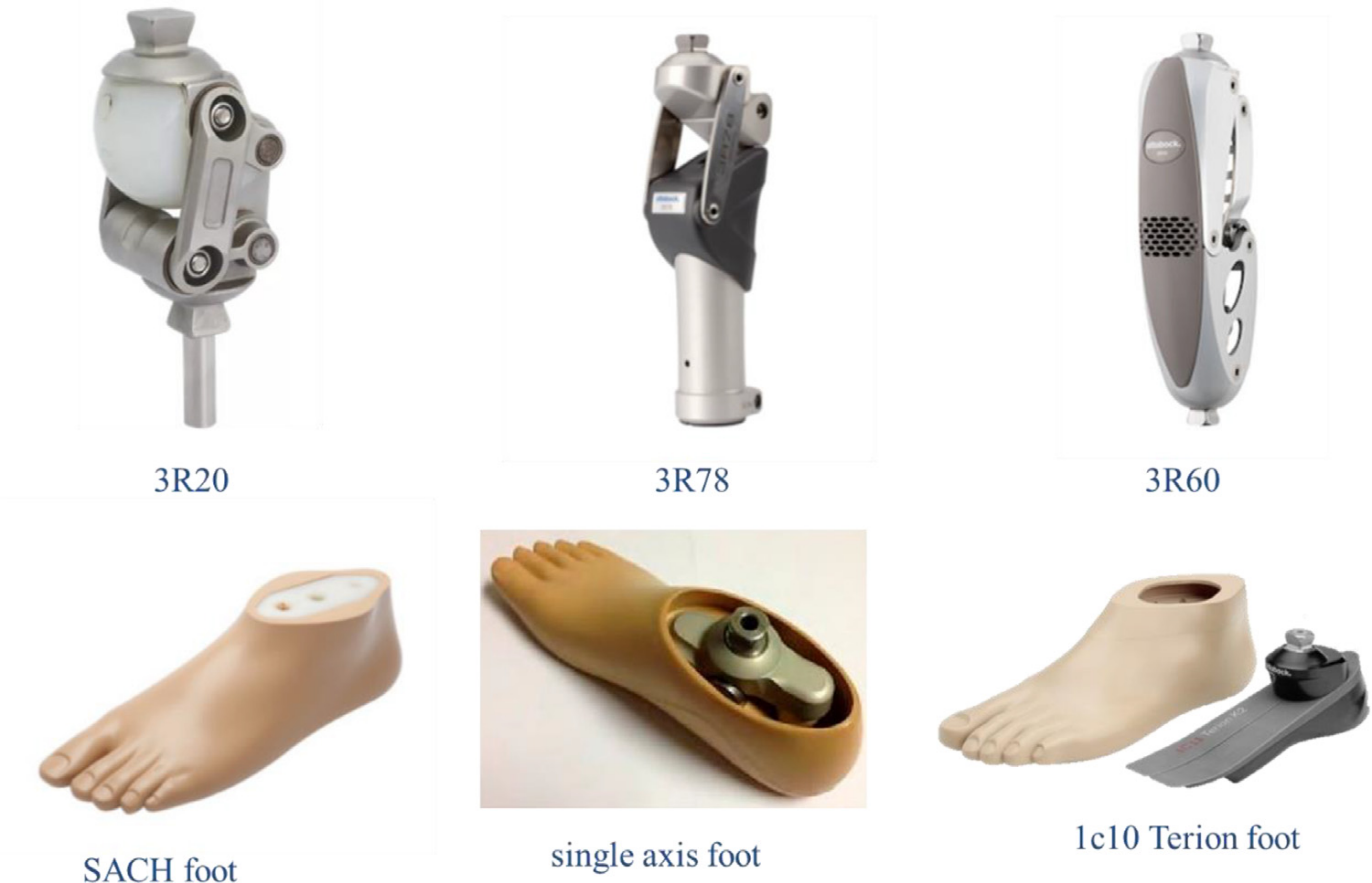
Passive prosthetic component used by participants.
2. File 02 spatio-temporal parameters.xlsx: Spatio-temporal parameters are in two sheets (*N* for healthy subjects and AMP for above knee participants). Each variable starts with the acronym AVE means that the value is the average of three gait cycles for the considered side (Left or right in healthy subjects or prosthetic and intact legs in above knee amputees. Fig. 2 shows the distance parameters that include step width, step length and stride length In meters, while temporal parameters (in seconds) include: gait cycle duration, stance phase duration, swing phase duration, double support duration, and speed. Table 1 shows these variables and their definitions.

**Fig. 2:**
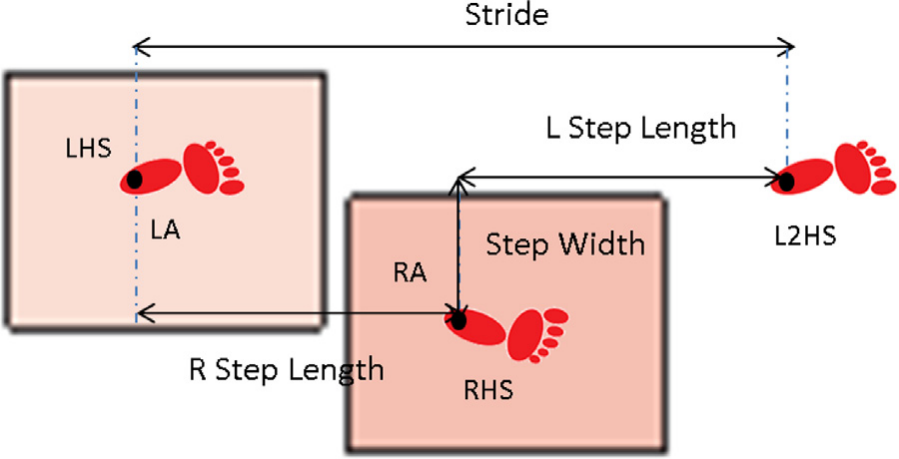
The distance parameters where the abbreviations mean: RA: right ankle, LA: left ankle, LHS means left heel strike, and L2HS means the next left heel strike [3].

**Table 1:** Spatio-temporal parameters definitions in File 02 spatio-temporal parameters.xlsx.

No.	Variables	Definition
1	Participant's code	It consists of Participant's group identifier where 'NS' mean healthy subjects while AMP means above knee amputee. The letters 'M' and 'F' code the participant's gender (Male or Female). While the number at the end of the participant's code identifies the subject according to gender group's.
2	AVE step width [m]	Transversal distance between the right and left ankle joint center [3,4].
3	AVE R/L step length [m]	Longitudinal distance from one ankle joint center during heel strike to the next ankle joint center heel strike of different foot [5].
4	AVE Gait Cycle Duration[s]	Total time that begins with Heel strike until the second heel strike of the same limb [6].
5	AVE R/L Stance Phase Duration [s]	Percentage of gait cycle that begins with heel strike and ends at toe-off of the same limb [5,7].
6	AVE R/L Swing Phase Duration [s]	The period during which the foot is in the air for the purpose of limb advancement [5,7].
7	AVE R/L Double Support Duration [s]	Time in which both feet are in contact with the floor [5,7].
8	AVE Speed [m/s]	The Mean velocity of progression equals stride length divided by gait cycle duration [5,7].

**Table 2:** Spreadsheets abbreviations and meanings in gait kinematic and kinetic files.

File Name	Spreadsheets in file	Acronym Meaning
File 03 lower limb joint angles in sagittal plane	N_AFE	Ankle Dorsiflexion-Plantarflexion angles of Normal subjects
	N_KFE	Knee Flexion-Extension angles of Normal subjects
	N_HIPFE	HIP Flexion-Extension angles of Normal subjects
	AMP_AFE	Ankle Dorsiflexion-Plantarflexion angles of above-knee amputee subjects
	AMP_KFE	Knee Flexion-Extension angles above-knee amputee subjects
	AMP_HIPFE	HIP Flexion-Extension angles of above-knee amputee subjects

File 04 ground reaction force components	N_GRF_Vertical	Ground Reaction Force Vertical component of Normal subjects
	N_GRF_AP	Ground Reaction Force Anterior-Posterior component of Normal subjects
	N_GRF_ML	Ground Reaction Force Medial-lateral component of Normal subjects
	AMP_GRF_Vertical	Ground Reaction Force Vertical component of above-knee amputee subjects
	AMP_GRF_AP	Ground Reaction Force Anterior-Posterior component of above-knee amputee subjects
	AMP_GRF_ML	Ground Reaction Force Medial-lateral component of above-knee amputee subjects

File 05 lower limb joint moments in sagittal plane	N_Moment_AFE	ankle Dorsiflexing-Plantar-flexing Moment of Normal subjects
	N_Moment_KFE	Knee Flexing- Extending Moment of Normal subjects
	N_Moment_HIPFE	HIP Flexing Extending Moment of Normal subjects
	AMP_Moment_AFE	Ankle Dorsiflexing-Plantar-flexing Moment of above-knee amputee subjects
	AMP_Moment_KFE	Knee Flexing- Extending Moment of above-knee amputee subjects
	AMP_Moment_HIPFE	HIP Flexing Extending Moment of above-knee amputee subjects
3. File 03 lower limb joint angles in sagittal plane.xlsx: three lower limb joint angles are calculated: ankle dorsiflexion-Plantarflexion AFE, knee flexion-extension KFE, and hip flexion-extension HIPFE. All angles in Fig. 3 were measured in degree and defined according to [1] where positive values mean flexion angle.

**Fig. 3:**
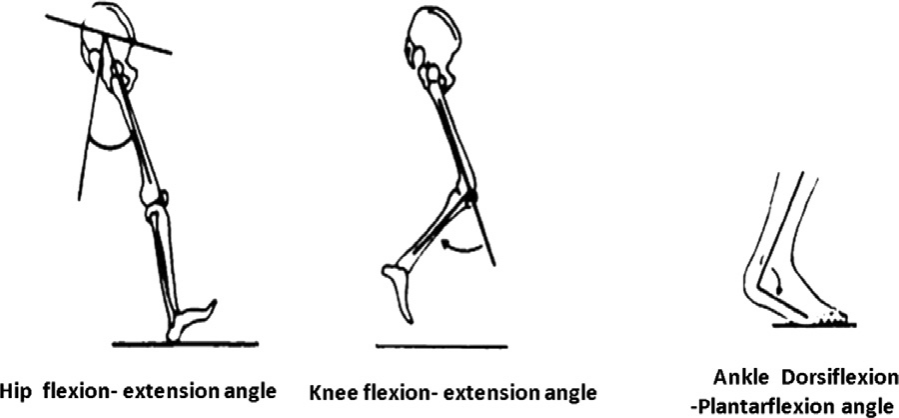
Lower limb joints angles in the sagittal plane.
4. File 04 ground reaction force components.xlsx contains three components of ground reaction force: the vertical, Anterior-Posterior AP, and Medial-lateral ML.They were measured using force plate, smoothed using piecewise cubic interpolation, and display as a percent of subject's body weight.
5. File 05 lower limb joint moments in sagittal plane.xlsx: three external moments in the sagittal plane are: ankle dorsiflexing-Plantar-flexing moment AFE, knee flexing-extending moment KFE, and hip flexing-extending moment HIPFE that all were measured in N.m/k, and defined according to [1] where positive values are flexing moments.

**Fig. 4:**
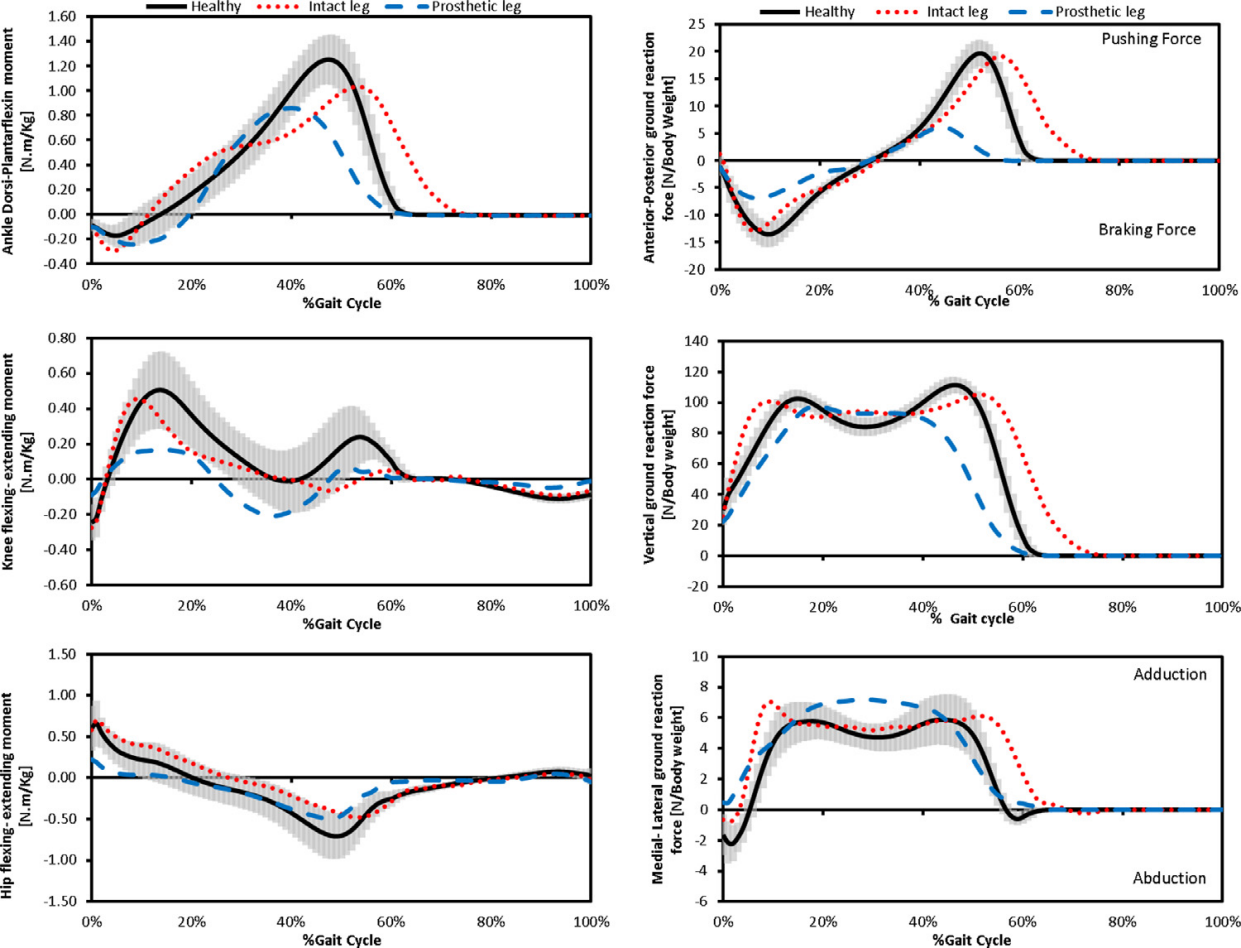
Values of ground reaction force components and lower limb joints moments in the sagittal plane for healthy subjects and both legs of above-knee amputees (intact and prosthetic).

Fig. 4 shows mean values of ground reaction force components and lower limb joints moments in the sagittal plane for healthy subjects surrounded by one standard deviation, in addition, mean values for both legs of above-knee amputees (intact and prosthetic).

## 2 Experimental Design, Materials and Methods

Gait analysis was conducted using six video cameras (BTS SMART-D system) for the kinematic analysis and two force plates (9281EA Kistler, Switzerland) for the kinetic analysis. The cameras and force plates were acquired data at 200Hz sampling rate.

34 subjects participated in this dataset:14 subjects (5 females- 9 males) with a unilateral above-knee amputation and 20 healthy subjects (10 females- 10 males). Healthy participants did not suffer from any health problems that affect walking. Unilateral above-knee amputees' inclusion criteria were:

1. They had unilateral amputations due to an injury (a war injury, an accident, or a surgical procedure following a congenital malformation).
2. Their residual limb free of pain and health problems.
3. They use their prosthesis for at least six months.
4. Hip joint moves along its entire range of motion.
5. They live within the community and walk without assistive devices. *K*- level for them one of the following:
  • K2: limited community ambulator.
  • K3: community ambulator.
  • K4: Typical of the prosthetic demands of the child, active adult, or athlete.

The experimental protocol followed biomedical engineering ethics and was approved by the biomedical engineering department at Damascus University- Syria.

**Subject preparation:**

**Fig. 5:**
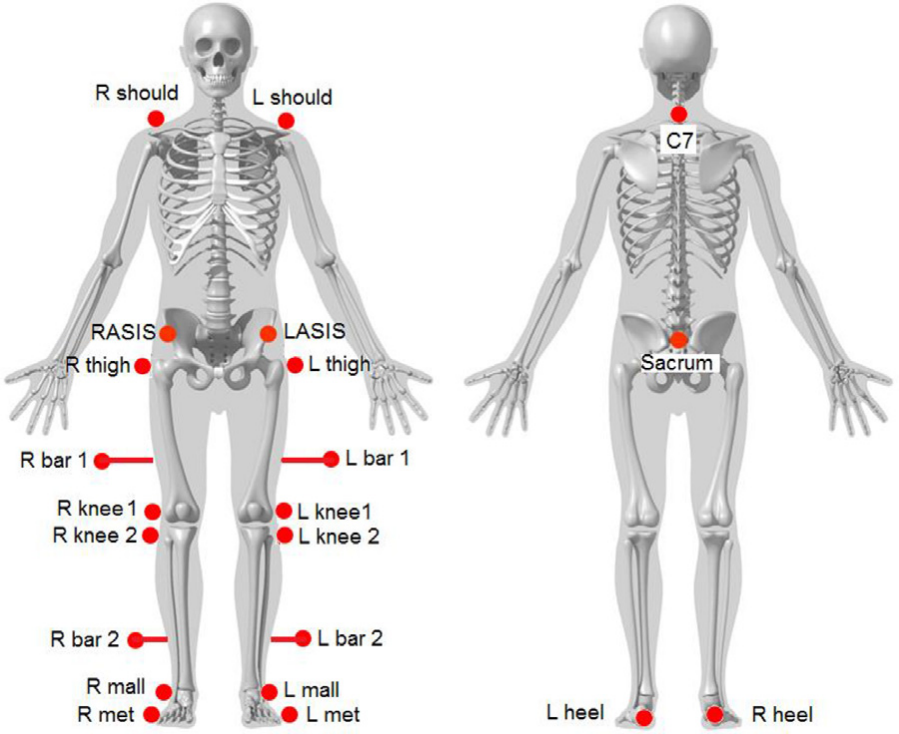
The positioning of 22 markers on the subject's body based on [1].

First, the participant gave verbal consent after explaining the details of the experiment and answering her/his questions. Then, researchers collected participant's personal information, as well as took her/his body measurements that included: weight, height, leg lengths, ASIS breadth, pelvis depth, knee diameter, and ankle width [1,8]. After that, the retro-reflective markers were adhesive to anatomical landmarks that were determined by palpation. Twenty-two markers were applied while the subject held an orthostatic position. Twenty of them are spherical (of which 4 are attached on a rigid bar), while the remaining two are hemispherical. Fig. 5 shows the positioning of twenty-two markers on the subject's body according to the following parts:

1. Three markers on the trunk: They were at the 7th cervical vertebra (c7), the right acromion (r should), and the left acromion (l should).
2 Three markers on the pelvis: one marker on each ASIS (r asis-l asis) and one marker on the back, in correspondence to the second sacral vertebra (sacrum).
3. Three markers on each thigh: one marker on the great trochanter (r thigh - l thigh), one on the lateral femoral condyle (r knee 1-l knee 1), and anther marker fixed on a lateral bar must be securely attached to the thigh using an adaptable strap (r bar 1-l bar 1). The bar must lie on the same plane defined by the virtual line between the hip joint center and the knee joint center, and the line between the medial and lateral femoral condyles (flexion-extension axis of the knee).
4. Three markers on each shank: one marker on the head of the fibula (r knee 2-l knee 2), which can be identified through palpation, one on the lateral malleolus (r mall-l mall), and one on a lateral bar (r bar 2-l bar 2) securely attached to the thigh using an adaptable strap.
5. Two markers on each foot: one marker on the fifth metatarsal head (r met-l met) and one hemispherical marker on the heel (r heel-l heel).

**Raw data acquisition:**

The subjects were asked to perform two different tasks while motion capture system acquiring data:

1. Standing task: the subject held an orthostatic position for at least 3 - 5 seconds on the top of the force platform. The feet of the subject must be aligned to avoid having one foot in a more anterior or posterior position with respect to the other.
2. Walking task: the subject walked normally in the straightest way possible across the working volume defined during the calibration phase of the optoelectronic system. The markers placed on the subject must be clearly within the field of view of the cameras during the whole acquisition. The subject must perform an entire stance phase of a single foot on one of the force platforms. The foot strike on the platform should be spontaneous.
3. The walking recording should be repeated 3–5 times.

**Gait analysis parameters calculation:**

Gait parameters were calculated using BTS Smart Clinic software. Each gait cycle was determined by specifying foot contact events [9]:

• Two initial contact events: the time when the heel marker trajectory reached the lowest vertical position.
• One Foot off event: the time of the lowest vertical position of the toe marker (met marker).

After that, gait parameters were automatically calculated according to equations in [1]. For each gait parameter, the average of three gait cycles was exported to the European MiFID Template (EMT) file since BTS software generated data in a format that needed a company software to open. Matlab code was used to import EMT files and to separate the data of interest arranged in excel files.

## Ethics Statement

Each participant was provided with information about the study and provide verbal consent. The study was approved by Biomedical Engineering Ethical Committee at biomedical engineering department at Damascus University prior to conducting the study.

## CRediT authorship contribution statement

**Rufaida Hussain:** Data curation, Writing – original draft. **Zouheir Marmar:** Supervision, Writing – review & editing.

## Declaration of Competing Interest

The authors declare that they have no known competing financial interests or personal relationships which have or could be perceived to have influenced the work reported in this article.
